# Exploring CD79b, LC3, and TERT expression in NON-GCB DLBCL: markers associated with Rituximab-Cyclophosphamide-Doxorubicin-Vincristin-Prednisone treatment response

**DOI:** 10.1007/s44313-026-00129-2

**Published:** 2026-05-11

**Authors:** Bethy S. Hernowo, Arnita Ilanur, Hasrayati Agustina, Amaylia Oehadian, Birgitta M. Dewayani, Anglita Yantisetiasti, Hermin A. Usman, Etis Primastari, Okky Husain, Nia Nuraeni, Nurvita Trianasari

**Affiliations:** 1 Department of Anatomical Pathology, Faculty of Medicine, Padjadjaran University/Dr. Hasan Sadikin General Hospital, Bandung, West Java Indonesia; 2 Department of Internal Medicine, Faculty of Medicine, Padjadjaran University/Dr. Hasan Sadikin General Hospital, Bandung, West Java Indonesia; 3 School of Economics and Business, Telkom University, Bandung, West Java Indonesia

**Keywords:** DLBCL non-GCB, CD79B, LC3, TERT, R-CHOP, Immunohistochemical markers

## Abstract

**Introduction:**

Diffuse large B-cell lymphoma (DLBCL) represents the most common subtype of non-Hodgkin lymphoma. The non-germinal center B-cell-like (non-GCB) subtype is associated with a poor prognosis and reduced response rates to rituximab cyclophosphamide-doxorubicin-vincristin-prednisone (R-CHOP) therapy. Resistance may involve cluster of differentiation 79B (CD79B)-driven B-cell receptor (BCR)/nuclear factor kappa B activation, microtubule-associated protein 1 light chain 3 (LC3)-mediated autophagy, and telomerase reverse transcriptase (TERT)-related survival signaling. The interrelationships between these markers and their associations with therapeutic responses remain unclear. This study evaluated the association between CD79B, LC3, and TERT expression and response to R-CHOP in non-GCB DLBCL.

**Methods:**

This analytical case–control study included 58 paraffin-embedded samples from patients with non-GCB DLBCL diagnosed at the Dr. Hasan Sadikin General Hospital, Bandung, Indonesia (2017–2023). Tumor characteristics were assessed using immunohistochemical staining for CD79B, LC3, and TERT. Expression levels were evaluated semi-quantitatively based on staining intensity and the proportion of positive tumor cells. Treatment responses were categorized as response (complete/partial) or non-response (stable/progressive). Statistical analyses were performed using the chi-square test, Fisher’s exact test, and binary logistic regression, with significance defined as *p* < 0.05.

**Results:**

TERT, CD79B, and LC3 expression was detected in 53.4%, 55.2%, and 62.1% of the cases, respectively. Among non-responsive patients, TERT and LC3 expression rates were 62.1% and 69.0% (*p* > 0.05), respectively. CD79B expression was significantly associated with non-response (*p* = 0.002). Moreover, multivariate analysis identified positive CD79B expression as an independent predictor of non-response to R-CHOP therapy (OR = 9.72).

**Conclusion:**

CD79B expression was independently associated with a poor response to R-CHOP therapy in non-GCB DLBCL. The positive association between TERT and LC3 suggests the presence of additional metabolic adaptation mechanisms supporting tumor survival.

## Introduction

Diffuse large B-cell lymphoma (DLBCL) is the most common and aggressive type of non-Hodgkin lymphoma (NHL) [1]. DLBCL is classified into two major subtypes based on the cell origin and genetic expression: *germinal center B-cell-like* (GCB) and *non-germinal center B-cell-like* (non-GCB). The Hans algorithm, based on immunohistochemistry, is widely employed in clinical practice to distinguish between the two subtypes [2]. The standard treatment for DLBCL consists of a combination regimen of rituximab, cyclophosphamide, doxorubicin, vincristine, and prednisone (R-CHOP). This regimen achieves a cure rate of 60–70%, but approximately 30–40% of patients still experience relapse or remain refractory to therapy [3]. Resistance is more frequently observed in the non-GCB subtype, which is associated with activation of survival pathways and impairment of apoptotic mechanisms [4].

Several molecular alterations have been implicated in the suboptimal therapeutic responses observed in patients with non-GCB DLBCL. Cluster of differentiation 79B (CD79B), a key signaling component of the B-cell receptor (BCR) complex, plays a crucial role in activating the classical nuclear factor kappa B activation (NF-κB) signaling pathway, thereby promoting lymphoma cell survival and proliferation [5].

Autophagy is a tightly regulated cellular recycling process that maintains metabolic homeostasis through lysosomal degradation of damaged organelles and misfolded proteins, typically assessed using markers, including microtubule-associated protein light chain 3 (LC3) and Beclin-1. In lymphoma, autophagy plays a dual context-dependent role, either inhibiting tumor growth or promoting survival under therapeutic stress. Recent evidence has indicated that increased autophagic flux contributes to chemoresistance in DLBCL by enabling tumor cells to evade apoptosis under cytotoxic conditions [6].

Telomerase reverse transcriptase (TERT), the catalytic subunit of telomerase, has emerged as a multifunctional regulator of tumor biology. In addition to its canonical role in telomere elongation, TERT exerts extra-telomeric functions that modulate oncogenic signaling pathways, including NF-κB, protein kinase B (AKT), and myelocytomatosis oncogene (MYC), thereby promoting cell proliferation, survival, and resistance to apoptosis. In B-cell malignancies, TERT contributes to immune evasion and genomic stability while sustaining the constitutive activation of pro-survival transcriptional programs. Elevated TERT expression and promoter mutations have been correlated with poor prognosis and therapeutic resistance in various cancers, indicating their potential involvement in treatment refractoriness [7].

Recent studies have suggested a biological link between BCR signaling, autophagy, and telomerase activity in lymphoma. Activation of BCR through CD79B stimulates key survival pathways such as NF-κB and PI3K/AKT. This activation can also induce autophagy, indicated by increased LC3 expression, which enables tumor cells to adapt to stress [8, 9]. Concurrently, TERT supports cell survival by enhancing mitophagy, a selective form of autophagy, through regulation of PTEN-induced kinase 1 (PINK1) and LC3 [10]. Together, these mechanisms preserve mitochondrial function and may contribute to chemotherapy resistance. Although the interplay between CD79B, LC3, and TERT has not yet been fully explored in non-GCB DLBCL, the overlap of their signaling pathways suggests that they may collectively support tumor progression and diminish treatment response. Therefore, this study aimed to analyze the association between the immunohistochemical (IHC) expression of CD79B, LC3, and TERT and the response to R-CHOP immunochemotherapy in patients with non-GCB DLBCL.

## Materials and methods

### Study design and samples

This study employed an analytical, observational, case–control design using paraffin-embedded tissue samples from patients diagnosed with non-GCB DLBCL. Samples were obtained from the Department of Anatomical Pathology at the Dr. Hasan Sadikin General Hospital, Bandung, Indonesia, between 2017 and 2023. Clinical data, including age, sex, disease stage, B symptoms, International Prognostic Index (IPI) score, and treatment response, were retrieved from medical records.

The inclusion criteria were histopathologically confirmed non-GCB DLBCL, as determined by the Hans algorithm using CD10, BCL6, and multiple myeloma oncogene 1 (MUM1) immunophenotyping; complete clinical data; and sufficient formalin-fixed, paraffin-embedded (FFPE) tissue available for IHC evaluation. Patients were excluded if tissue preservation was inadequate or if data on R-CHOP therapy were incomplete.

From the total source population of 137 patients with DLBCL, 81 had complete data available. Based on the minimum sample size, 29 responders and 29 non-responders were selected. Therefore, balanced groups reflect a sample-size-driven selection strategy rather than real-world response proportions.

### Histopathological and IHC examination

Histopathological examination was performed on 4-µm sections of FFPE tissues stained with hematoxylin and eosin to confirm the diagnosis of non-GCB DLBCL subtype, as determined by the Hans algorithm.

IHC staining was performed on serial sections using three different monoclonal antibodies: anti-CD79B (clone SP18; Thermo Fisher Scientific, USA), anti-LC3B (clone EPR18709; Abcam), and anti-TERT (clone ab183105; Abcam). All procedures were performed according to the manufacturers’ protocols and optimized at the Department of Anatomical Pathology, Faculty of Medicine, Universitas Padjadjaran. Appropriate positive and negative controls were incorporated into each staining run.

CD79B antibody was utilized to evaluate membranous and/or cytoplasmic expression in neoplastic B-cells, whereas LC3 antibody was used to assess cytoplasmic granular autophagy-related expression. The TERT antibody was employed to assess the cytoplasmic expression in tumor cells, reflecting its role as the catalytic subunit of telomerase, which is involved in chromosomal maintenance and cell survival.

### Interpretation of IHC staining

The IHC results were independently evaluated by two anatomical pathologists blinded to the clinical outcomes. Additionally, any discrepancies were resolved by consensus.

For CD79B evaluation, the staining intensity was graded as 0 (negative; no staining), 1 (weak; light yellow), 2 (moderate; brown-yellow), or 3 (strong; dark brown). The proportion of positive tumor cells was scored as 0 (0%), 1 (5–25%), 2 (26–50%), 3 (51–75%), or 4 (> 75%) based on membranous or cytoplasmic staining. The final immunoreactivity score (IRS) was calculated by summing the intensity and proportion scores, yielding a total score ranging from 0 to 7. Tumors with scores < 6 were defined as negative (low expression), whereas those with scores ≥ 6 were considered positive (high expression) [11].

For LC3 evaluation, cytoplasmic granular staining in tumor cells was assessed semi-quantitatively, considering both the proportion of positive cells and staining intensity, as described by Zhang et al. (2018). The proportion of LC3-positive tumor cells was scored as 0 (0–5%), 1 (6–25%), 2 (26–50%), 3 (51–75%), or 4 (76–100%). Furthermore, the staining intensity was graded as 0 (negative), 1 (weak; light yellow), 2 (moderate; tan), and 3 (strong; brown). The LC3 IRS was calculated by multiplying the percentage score by the intensity score, resulting in a total score ranging from 0 to 12. Tumors with an IRS of 0–6 were classified as negative, whereas those with a score of 7–12 were considered positive. All slides were reviewed independently by two pathologists under a light microscope (400 × magnification) to confirm the staining distribution and consistency in intensity [12].

For TERT evaluation, the cytoplasmic staining of tumor cells was assessed semi-quantitatively using a combined intensity and percentage scoring system, as described by Su et al. (2023). Staining intensity was graded as 0 (negative), 1 (weak), 2 (moderate), or 3 (strong); meanwhile, the proportion of positive tumor cytoplasm was scored as 0 (0%), 1 (< 10%), 2 (10–50%), 3 (51–80%), or 4 (> 80%). The final IRS was calculated by multiplying the intensity and distribution scores, resulting in a total score ranging from 0 to 12. Based on previous studies and validation cutoffs, scores of 0–4 were considered negative, whereas scores ≥ 6 were classified as positive (strong expression) [13].

### Treatment response assessment

Response assessment was performed according to the Lugano 2014 criteria using contrast-enhanced computed tomography imaging (not positron emission tomography [PET]/CT) at least four weeks after the completion of six cycles of R-CHOP. Imaging results were interpreted based on routine clinical radiology reports and were not centrally reviewed. Patients were categorized as responders (complete or partial remission) or non-responders (stable or progressive disease).

### Statistical analysis

All statistical analyses were performed using SPSS version 26.0 (IBM Corp., Armonk, NY, USA). Associations between categorical variables were analyzed using the chi-squared test. Non-parametric data comparisons were conducted using the Mann–Whitney U test. Logistic regression analysis was performed to identify independent predictors of treatment response. Results were expressed as odds ratios (OR) with 95% confidence intervals (CI), and *p*-values < 0.05 were considered statistically significant.

### Ethical considerations

This study was approved by the Research Ethics Committee of the Faculty of Medicine at Universitas Padjadjaran, Bandung, Indonesia (approval number 055/UN6). KEP/EC/2025 dated June 23, 2025).

## Result

### Patient characteristics

The study analyzed 58 patients with non-GCB DLBCL who were treated with R-CHOP. The clinical and pathological features are summarized in Table 1. The mean age was 53.2 years, with 79.3% of patients aged 60 years or younger. Of the patients, 53.4% males and 46.6% females. Extranodal involvement was observed in 77.6% of cases, while 22.4% presented with nodal disease. Most patients had early-stage disease (stages I–II, 74.2%) and low IPI scores (0–1, 87.9%). All cases were classified as non-GCB using the Hans algorithm, characterized by universal CD10 negativity (100%) and high MUM1 expression (89.7%). Moreover, BCL6 positivity was observed in 24.1% of the cases.

**Table 1:** Patient characteristics overview

Variable	*N* = 58
**Age**	
Mean ± Std	53.16 ± 10.397
Median	51.00
Range (min–max)	27.00–78.00
< 60 years	46(79.3%)
> = 60 years	12(20.7%)
**Gender**	
Male	31(53.4%)
Female	27(46.6%)
**BMI**	
Underweight	2(3.4%)
Normoweight	42(72.4%)
Overweight	12(20.7%)
Obesity	2(3.4%)
**Clinical symptoms**	
Present	27(46.6%)
Absent	31(53.4%)
**Tumor Location**	
Nodal	13(22.4%)
Extranodal	45(77.6%)
**IPI Score**	
0	37(63.8%)
1	14(24.1%)
2	7(12.1%)
**Stage**	
I	19(32.8%)
II	24(41.4%)
III	7(12.1%)
IV	8(13.8%)
**BCL6**	
Positive	14(24.1%)
Negative	44(75.9%)
**MUM1**	
Positive	52(89.7%)
Negative	6(10.3%)
**CD10**	
Positive	0(0.0%)
Negative	58(100.0%)
**CD79b**	
Positive	32(55.2%)
Negative	26(44.8%)
**LC3**	
Positive	36(62.1%)
Negative	22(37.9%)
**TERT**	
Positive	31(53,4%)
Negative	27(46,6%)

Categorical data is presented as number/frequency and percentage, while numerical data is presented as mean, median, standard deviation, and range

Patient characteristics according to R-CHOP treatment response are presented in Table 2. Twenty-nine patients (50%) achieved a complete or partial response (responders), while 29 (50%) demonstrated stable or progressive disease (non-responders). None of the clinical parameters, including age, sex, body mass index, stage, IPI, or primary tumor site, exhibited a statistically significant association with treatment response (*p* > 0.05).

**Table 2:** Comparison of research patient characteristics based on treatment response

Variabel	Respon Terapi		OR CI 95%	Nilai P
	Tidak Respon	Respon		
	*N* = 29	*N* = 29		
**Age**				0.088
Mean ± Std	55.48 ± 11.344	50.83 ± 8.953		
Median	54.00	50.00		
Range (min–max)	27.00–78.00	36.00–71.00		
**Age Category**			3.900	0.052
> = 60 years	9(31.0%)	3(10.3%)	(0.933–16.310)	
< 60 years	20(69.0%)	26(89.7%)		
**Gender**			1.518	0.430
Male	17(58.6%)	14(48.3%)	(0,538–4,284)	
Female	12(41.4%)	15(51.7%)		
**BMI**			**-**	0.591
Underweight	1(3.4%)	1(3.4%)		
Normoweight	22(75.9%)	20(69.0%)		
Overweight	5(17.2%)	7(24.1%)		
Obesity	1(3.4%)	1(3.4%)		
**Clinical symptoms**			2.692	0.065
Present	17(58.6%)	10(34.5%)	(0.929–7.801)	
Absent	12(41.4%)	19(65.5%)		
**Tumor Location**			0.356	0.115
Nodal	4(13.8%)	9(31.0%)	(0.095–1.326)	
Extranodal	25(86.2%)	20(69.0%)		
**IPI Score**			**-**	0.084
0	15(51.7%)	22(75.9%)		
1	10(34.5%)	4(13.8%)		
2	4(13.8%)	3(10.3%)		
**Stage**			**-**	0.378
I	7(24.1%)	12(41.4%)		
II	15(51.7%)	9(31.0%)		
III	2(6.9%)	5(17.2%)		
IV	5(17.2%)	3(10.3%)		
**BCL6**			0.304	0.066
Positive	4(13.8%)	10(34.5%)	(0.083–1.120)	
Negative	25(86.2%)	19(65.5%)		
**MUM1**			1.000	1.000
Positive	26(89.7%)	26(89.7%)	(0.184–5.420)	
Negative	3(10.3%)	3(10.3%)		
**CD10**			**-**	1.000
Positive	0(0.0%)	0(0.0%)		
Negative	29(100.0%)	29(100.0%)		

For ordinal data, p-values were calculated using the Mann–Whitney U test. For categorical variables, *p*-values were determined using the chi-square test. Statistical significance value was defined as a *p*-value < 0.05

### Immunoexpression of CD79B, LC3, and TERT

The expression profiles of CD79B, LC3, and TERT in relation to R-CHOP treatment response are summarized in Table 3.

**Table 3:** Comparison of CD79B, LC3, and TERT expression in patients with non-GCB DLBCL according to treatment response

Variable	Treatment Response		OR CI 95%	*p*-value
	Response	Non-response		
	***N*** = 29	***N*** = 29		
**CD79b**			5.971	0.002*
Positive	22(75.9%)	10(34.5%)	(1.901–18.754)	
Negative	7(24.1%)	19(65.5%)		
**LC3**			1.806	0.279
Positive	20(69.0%)	16(55.2%)	(0.617–5.287)	
Negative	9(31.0%)	13(44.8%)		
**TERT**			2.014	0.188
Positive	18(62.1%)	13(44.8%)	(0.706–5.744)	
Negative	11(37.9%)	16(55.2%)		

For categorical data, *p*-values were calculated using the chi-squared test. The significance value was based on a *p*-value < 0.05

**Fig. 1:**
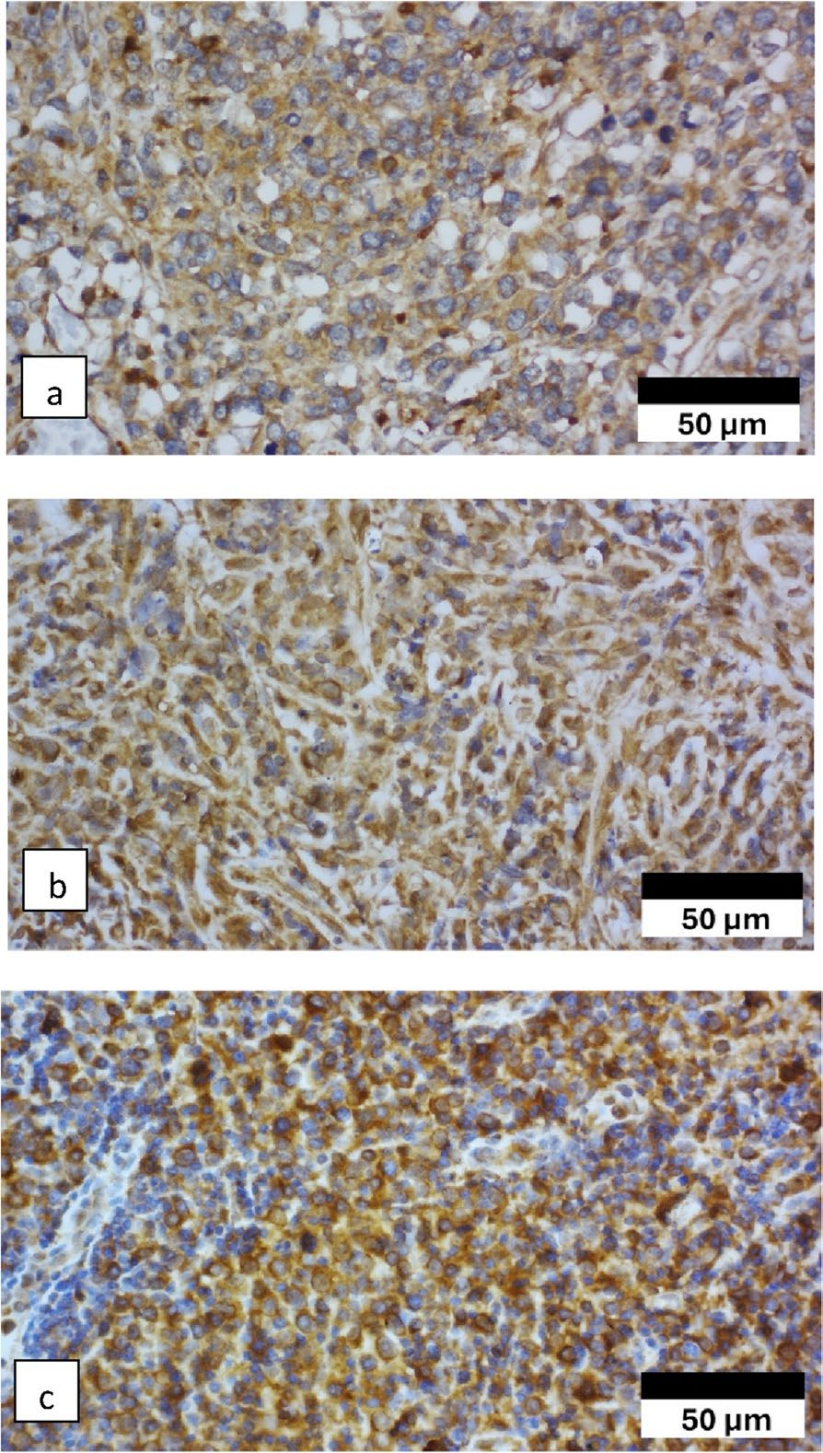
Cluster of differentiation 79B (CD79B) immunoexpression localized to the cell membrane. **a** Weak CD79b immunoexpression (400 × magnification). **b** Moderate CD79b immunoexpression (400 × magnification). **c** Strong CD79b immunoexpression (400 × magnification)

CD79B expression was positive in 32 of the 58 patients (55.2%). The response group demonstrated a reduced frequency of CD79B positivity (34.5%) compared to that observed in the non-response group (75.9%) (*p* = 0.002), indicating a significant association between CD79B expression and R-CHOP response (Table 3). Representative CD79B IHC staining is illustrated in Fig. 1.

**Fig. 2:**
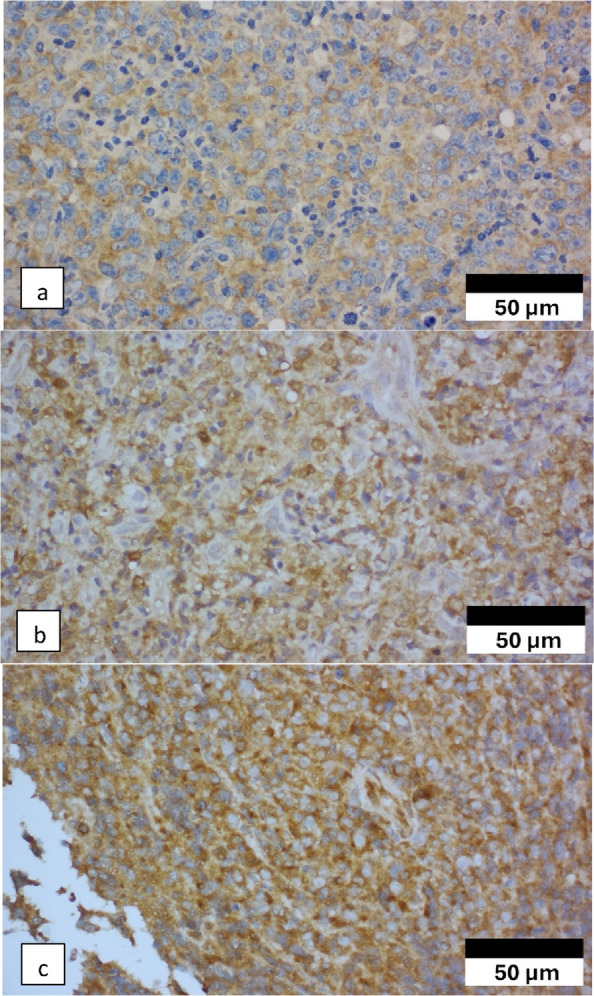
Microtubule-associated protein 1 light chain 3 (LC3) immunoexpression. **a** Weak LC3 immunoexpression (400 × magnification). **b** Moderate LC3 immunoexpression (400 × magnification). **c** Strong LC3 immunoexpression (400 × magnification)

LC3 expression was positive in 36 of 58 cases (62.1%). Moreover, LC3 positivity was observed in 55.2% of the response group and 69.0% of the non-response group, but the difference was not statistically significant (*p* = 0.279) (Table 3). Figure 2 depicts representative LC3 IHC findings.

**Fig. 3:**
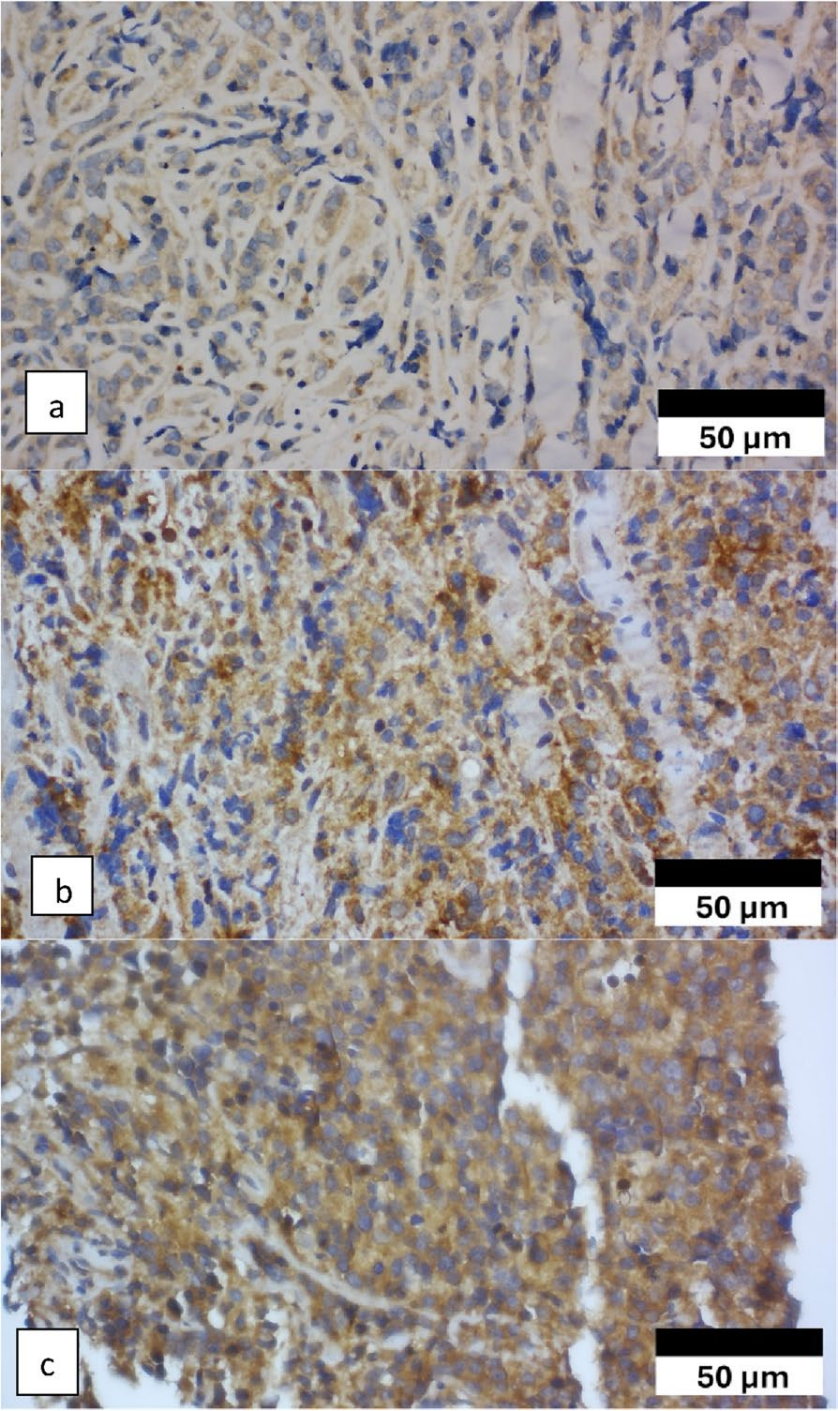
Telomerase reverse transcriptase (TERT) immunoexpression. **a** TERT immunoexpression (400 × magnification). **b** Moderate TERT immunoexpression (400 × magnification). **c** Strong TERT immunoexpression (400 × magnification)

TERT expression was positive in 31 of the 58 patients (53.4%). The response group had a lower TERT positivity (44.8%) than the positivity noted in the non-response group (62.1%); however, the difference was not statistically significant (*p* = 0.188) (Table 3). Figure 3 demonstrates representative images of TERT expression.

### Correlation between markers

TERT and LC3 expression levels were significantly positively correlated (*p* = 0.002). LC3-positive cases had a higher TERT-positivity rate (69.4%) than LC3-negative cases (27.3%), suggesting that TERT expression tends to increase in tumors with LC3 positivity (Table 4).

**Table 4:** Comparison between CD79b and TERT with LC3

Variable	LC3		OR CI 95%	*p*-value
	Positive	Negative		
	***N*** = 36	***N*** = 22		
**CD79b**			2.556	0.088
Positive	23(63.9%)	9(40.9%)	(0.861–7.590)	
Negative	13(36.1%)	13(59.1%)		
**TERT**			6.061	0.002*
Positive	25(69.4%)	6(27.3%)	(1.870–19.647)	
Negative	11(30.6%)	16(72.7%)		

For categorical data, *p*-values were calculated using the chi-squared test. The significance value was based on a *p*-value < 0.05

CD79B positivity was observed in 64.9% of the LC3-positive cases and 38.9% of the LC3-negative cases; however, the difference was not statistically significant (*p* = 0.088) (Table 4).

No significant correlation was observed between CD79B and TERT expressions. CD79B positivity was detected in 61.3% of TERT-positive cases and 48.1% of the TERT-negative cases (*p* = 0.315) (Table 5).

**Table 5:** Comparison between TERT and CD79b

Variable	CD79b		OR CI 95%	*p*-value
	Positive	Negative		
	***N* = 32**	***N* = 26**		
**TERT**			1.705	0.315
Positif	19(59,4%)	12(46,2%)	(0.600–4.849)	
Negatif	13(40,6%)	14(53,8%)		

For categorical data, p-values were calculated using the chi-squared test. Statistical significance value was defined as a *p*-value < 0.05

### Multivariate logistic regression analysis

Multivariate logistic regression analysis identified CD79B positivity as independently associated with non-response to R-CHOP therapy (OR = 9.72, 95% CI = 2.29–41.19, *p* = 0.002). Age ≥ 60 years was also independently associated with non-response (OR = 8.78, 95% CI = 1.41–54.90, *p* = 0.020). In contrast, BCL6 positivity demonstrated a protective association, correlating with lower odds of non-response (OR = 0.17, 95% CI = 0.03–0.96, *p* = 0.045). However, TERT expression was not independently associated with treatment outcomes (OR = 0.73, 95% CI = 0.19–2.81, *p* = 0.648). Detailed results of the multivariate logistic regression analysis are presented in Table 6.

**Table 6:** Multivariate analysis

Variabel		B	S.E	Nilai P	OR	OR CI 95%	
						Lower	Upper
**Initial model**	1. Age Category > = 60	1,479	1,167	0,205	4,388	0,445	43,24
	2. CD79b positive	2,353	0,779	0,003	10,521	2,286	48,412
	3. IPI Score	−0,564	0,674	0,403	0,569	0,152	2,132
	4. Present clinical symptoms	1,368	0,71	0,054	3,928	0,977	15,79
	5. BCL 6 Positive	−1,725	0,927	0,063	0,178	0,029	1,097
	6. TERT Positive	0,313	0,687	0,648	1,368	0,356	5,258
**Final Model**	1. Age Category > = 60	2,173	0,935	0,020	8,784	1,406	54,896
	2. CD79b positive	2,274	0,737	0,002	9,716	2,292	41,189
	3. Present clinical symptoms	1,35	0,7	0,054	3,857	0,977	15,222
	4. BCL 6 Positive	−1,793	0,896	0,045	0,166	0,029	0,964

Multivariate analysis with binary logistic regression

Among the analyzed variables, CD79B positivity exhibited the strongest association with non-response, followed by advanced age (≥ 60 years). These findings indicated that CD79B protein expression was most strongly associated with the treatment response category in patients with non-GCB DLBCL managed with R-CHOP.

## Discussion

We evaluated CD79B, LC3, and TERT expression in patients with non-GCB DLBCL and their association with R-CHOP response. Although CD79B mutations have been associated with adverse outcomes in previous genomic studies, our findings specifically reflected CD79B protein expression as assessed by IHC. LC3 and TERT expression were significantly common in the non-response group, but were not statistically significant in multivariate analysis. Additionally, TERT and LC3 expressions exhibited a significant positive correlation, indicating coordinated expression.

CD79B expression was significantly associated with non-response to R-CHOP therapy, supporting its role in the pathogenesis of non-GCB DLBCL. CD79B encodes a key immunoreceptor component of the BCR complex, which amplifies BCR signaling and activates downstream NF-κB and PI3K pathways involved in tumor survival. Genetic and molecular profiling studies have demonstrated that CD79B mutations and overexpression are common in activated B-cell-like or non-GCB subtypes, where they frequently co-occur with myeloid differentiation primary response 88 (MYD88) mutations and drive chronic active BCR signaling, contributing to inferior clinical outcomes [14]. Although CD79B mutations have been associated with adverse outcomes in previous genomic studies, our findings specifically reflected CD79B protein expression as assessed by immunohistochemistry. Recent studies have provided additional evidence of this association. CD79B overexpression correlates with R-CHOP response and is a potential target for polatuzumab vedotin therapy [15]. Patients with CD79B mutations have worse overall survival despite standard immunochemotherapy [16]. These findings suggest that CD79B-mediated BCR activation contributes to poor treatment response, consistent with our results.

LC3 expression was more common in the non-response group than in the response group, although the difference was not statistically significant. This suggests a link between increased autophagic activity and reduced sensitivity to R-CHOP. Similar findings were reported by Zhou et al. (2022), who identified an autophagy-related gene signature that predicts survival and treatment resistance in DLBCL. Patients with high autophagy-related gene expression had poor outcomes and increased activation of doxorubicin-resistance and NF-κB pathways [17]. Zhang et al. (2024) also demonstrated that reduced autophagy levels were correlated with shorter progression-free survival in DLBCL, highlighting the importance of balanced autophagic regulation in treatment response [18]. Mandhair et al. (2024) reported subtype-specific autophagic activity in DLBCL, suggesting that autophagy exerts distinct biological roles across different molecular subtypes [19]. These findings suggest a possible association between increased autophagy-related marker expression and treatment response. However, LC3 immunohistochemistry represents a static marker and does not directly reflect autophagic flux. The higher frequency of LC3 positivity in the non-response group aligns with the concept that dysregulated autophagy may enable tumor cells to evade chemotherapy-induced stress, thereby contributing to poor outcomes.

TERT expression was more common in the non-response group than in the response group, suggesting that increased activity may contribute to a poor response to R-CHOP therapy in non-GCB DLBCL. Moreover, TERT maintains telomere length and promotes cellular immortality. In addition, TERT exerts extratelomeric functions, including transcriptional regulation, DNA repair, mitochondrial protection, and modulation of oncogenic signaling pathways, thereby enhancing tumor survival and therapy resistance [7]. Genomic evidence supports the prognostic significance of telomere-related genes (*TRGs*) in patients with DLBCL. Zhao et al. (2023) demonstrated that higher *TRG* expression, including that of TERT-associated genes, is linked to poor survival and immune evasion [20]. Lam et al. (2016) demonstrated that TERT activation in B-cell lymphomas occurs primarily through epigenetic or transcriptional mechanisms, rather than through promoter mutations. These results suggest that aberrant TERT activation promotes lymphoma cell survival under chemotherapy-induced stress [21]. However, few studies have specifically assessed TERT protein expression and its functional implications in DLBCL. Future research integrating IHC, molecular, and functional assays is needed to confirm TERT's mechanistic role in DLBCL and its potential as a therapeutic target or predictive biomarker for the R-CHOP response.

TERT expression was positively correlated with LC3 expression (*p* = 0.002), suggesting a potential interaction between telomerase activity and autophagy, both of which may enhance tumor adaptation to cytotoxic stress. Furthermore, TERT regulates autophagy by modulating LC3 expression. In vascular and endothelial models, Hughes et al. (2021) demonstrated that TERT overexpression increased the conversion of LC3-I to LC3-II, a hallmark of autophagy activation. Inhibition of TERT reduces LC3 conversion and autophagic flux. Autophagy functions downstream of telomerases, forming a coordinated pathway that preserves cellular homeostasis and enhances resistance to oxidative stress [22]. Park et al. (2022) confirmed that TERT activation restores autophagic flux and LC3 processing in human arterioles, demonstrating that telomerase and autophagy work together to maintain nitric oxide–dependent vascular function under stress [23]. The findings support the correlation between TERT and LC3 expression observed in this study. TERT overexpression may potentiate autophagy-mediated survival signaling in DLBCL cells, contributing to non-response to R-CHOP therapy. The significant TERT–LC3 association may indicate a survival mechanism whereby telomerase activation and autophagy cooperate to sustain tumor viability during R-CHOP therapy.

Multivariate analysis identified CD79B as the primary independent marker associated with non-response to R-CHOP therapy, underscoring its key role in mediating resistance via sustained BCR-driven signaling. LC3 and TERT did not emerge as independent predictors, but likely contributed indirectly through metabolic and survival crosstalk, reflecting adaptive mechanisms that support lymphoma cell persistence under cytotoxic stress.

This study offers novel insights into treatment response–related biological processes in non-GCB DLBCL by evaluating CD79B, LC3, and TERT protein expression within the same patient cohort. Our integrated approach highlights the potential interplay between BCR signaling, autophagy-related pathways, and telomerase-associated processes, providing a more comprehensive understanding of the factors associated with the response to R-CHOP therapy. The use of well-characterized samples and standardized IHC evaluations further strengthened the robustness of our findings.

This study had certain limitations. The relatively small sample size may have limited the statistical power, particularly for LC3 and TERT, and may have contributed to the wide CIs in the multivariable analysis. The retrospective, single-center design and intentional sample size–driven selection strategy may have introduced selection bias and limited generalizability. In addition, significant molecular prognostic factors such as double-hit/double-expressor status, MYD88 mutations, and other genetic alterations were not available, raising the possibility of residual confounding. Furthermore, this study evaluated protein expression solely by immunohistochemistry, precluding functional or mechanistic inferences. The absence of survival endpoints analysis further limits the interpretation to associations with the treatment response category. Larger multicenter studies incorporating comprehensive molecular profiling and functional analyses are warranted to elucidate the biological roles of CD79B, LC3, and TERT in non-GCB DLBCL.

## Conclusion

CD79B protein expression was independently associated with non-response to R-CHOP therapy in patients with non-GCB DLBCL, underscoring its potential relevance in treatment response–related BCR/NF-κB signaling activity. Although LC3 and TERT expression were not independently associated with treatment outcomes, their positive correlation suggested a possible interplay between autophagy- and telomerase-associated processes in tumor cell survival during chemotherapy. These findings support a multifactorial biological context underlying the treatment response in non-GCB DLBCL and warrant further investigation in larger cohorts and functional studies.

## Data Availability

No datasets were generated or analysed during the current study.
